# A Human CD68 Promoter-Driven Inducible Cre-Recombinase Mouse Line Allows Specific Targeting of Tissue Resident Macrophages

**DOI:** 10.3389/fimmu.2022.918636

**Published:** 2022-07-06

**Authors:** Agata N. Rumianek, Ben Davies, Keith M. Channon, David R. Greaves, Gareth S. D. Purvis

**Affiliations:** ^1^Sir William Dunn School of Pathology, University of Oxford, Oxford, United Kingdom; ^2^Wellcome Trust Centre for Human Genetics, University of Oxford, Oxford, United Kingdom; ^3^Division of Cardiovascular Medicine, Radcliffe Department of Medicine, John Radcliffe Hospital, University of Oxford, Oxford, United Kingdom

**Keywords:** macrophage, Cre/*loxP*, inducible, hCD68, targeting, LysM.

## Abstract

Current genetic tools designed to target macrophages *in vivo* often target cells from all myeloid lineages. Therefore, we sought to generate a novel transgenic mouse which has a tamoxifen inducible Cre-recombinase under the control of the human CD68 promoter (hCD68-CreERT2). To test the efficiency and specificity of the of Cre-recombinase activity we crossed the hCD68-CreERT2 mice with a loxP-flanked STOP cassette red fluorescent protein variant (tdTomato) mouse. We established that orally dosing mice with 2 mg of tamoxifen for 5 consecutive days followed by a 5-day induction period resulted in robust expression of tdTomato in CD11b^+^ F4/80^+^ tissue resident macrophages. Using this induction protocol, we demonstrated tdTomato expression within peritoneal, liver and spleen macrophages and blood Ly6C^low^ monocytes. Importantly there was limited or no inducible tdTomato expression within other myeloid cells (neutrophils, monocytes, dendritic cells and eosinophils), T cells (CD4^+^ and CD8^+^) and B cells (CD19^+^). We also demonstrated that the level of tdTomato expression can be used as a marker to identify different populations of peritoneal and liver macrophages. We next assessed the longevity of tdTomato expression in peritoneal macrophages, liver and splenic macrophages and demonstrated high levels of tdTomato expression as long as 6 weeks after the last tamoxifen dose. Importantly, hCD68-CreERT2 expression is more restricted than that of LysM-Cre which has significant expression in major myeloid cell types (monocytes and neutrophils). To demonstrate the utility of this novel macrophage-specific Cre driver line we demonstrated tdTomato expression in recruited CD11b^+^CD64^+^F4/80^+^ monocyte-derived macrophages within the atherosclerotic lesions of AAV8-mPCSK9 treated mice, with limited expression in recruited neutrophils. In developing this new hCD68-CreERT2 mouse we have a tool that allows us to target tissue resident macrophages, with the advantage of not targeting other myeloid cells namely neutrophils and inflammatory monocytes.

## 1 Introduction

Tissue resident macrophages play an important sentinel role, maintaining tissue homeostasis by surveying local tissue niches for pathogen, and regulating the local inflammatory response (1, 2). Macrophages, also play an important role in initiating a programme of wound repair following infection or tissue injury (3). If tissue homeostasis is not fully restored and inflammation resolution not achieved macrophages can promote continued tissue damage and chronic inflammation in diseases such as diabetes, rheumatoid arthritis and atherosclerosis (4–6).

Tissues harbour two main macrophage populations; those seeded during embryonic development and those that are recruited *via* the circulation and are monocyte derived (7). Macrophages are classically defined by flow cytometry as having high expression of CD11b and F4/80, however, the levels of F4/80 between tissues varies considerably. For example, peritoneal macrophages express F4/80 at high levels, whereas capsular macrophages in the liver have relatively low levels of F4/80. This makes identifying tissue-resident macrophages difficult (8), especially if they are not under steady state (9). This problem has been further complicated by large scale sequencing projects that have identified increased heterogeneity within cell surface defined macrophage population (10–12).

Cell surface markers are readily available to discriminate common myeloid cell types such as neutrophils and monocytes from macrophages or dendritic cells in both the mouse and human, however, genetic tools for targeting macrophages are lacking in specificity (13). This is most likely due to the common haemopoietic progenitors which give rise to the common myeloid progenitor which produces neutrophils and monocytes (14), which are the cells that are mostly frequently targeted in so called macrophage specific mouse lines. A common progenitor has been identified for dendritic cells and macrophages which makes discrimination of these two related cells types challenging (15).

More complexity arises in the fact that tissue resident macrophages are thought to be a self-renewing population; however, there is now a bulk of evidence to suggest that with age embryonically seeded tissue resident macrophages are replenished by monocyte-derived macrophages (12, 16). This raises the question as to what a tissue resident macrophage actually is, and what their functions are in different tissues (17, 18). Within the heart tissue resident macrophages have essential and diverse functionality in health; for example in coronary blood and lymphatic vessel development (19, 20) and facilitation of cardiac conduction (21). They also have a critical role in tissue remodelling post injury following myocardial ischaemia (22). In other organs such as skeletal muscle, the eye and the pancreas it is becoming evident that tissue resident macrophages also have unique functions (23, 24). Additionally, within the peritoneal cavity, there is a homogenous population of CD11b and F4/80-expressing peritoneal macrophages but two populations can be identified by size. The large peritoneal macrophages (LPM) which account for approximately 90% of all peritoneal macrophages and are derived embryonically, and small peritoneal macrophages (SPM) which account for around 10% and are derived from circulating monocytes (25).

Human CD68 and its murine homolog, macrosialin (Cd68), are glycosylated type 1 transmembrane proteins that belong to the lysosomal/endosomal-associated membrane glycoprotein (LAMP) family and are widely used as a macrophage marker (26). Although widely cited to be localised to the endocytic compartment its function is not fully elucidated but roles in both antigen processing and as a scavenger receptor have been reported. The murine Cd68 (macrosalin) and human CD68 gene differ in that the first intron (IVS-1) of human CD68 gene region has an additional regulatory element which specifically directs CD68 expression in macrophages, whereas the first intron of the mouse Cd68 gene can direct expression on non-macrophage cells (27, 28). We have previously generated a transgenic reporter mouse line, by expressing a green fluorescent protein gene under the transcriptional control of a hCD68-promoter IVS-1 expression cassette (29). This directs expression of GFP in bone marrow, circulating monocytes and tissue resident and recruited macrophages (30). There is also considerable expression in neutrophils and other myeloid cell linages due the expression of the hCD68-GFP transgene during embryogenesis and haematopoiesis (29).

To better study tissue resident macrophage biology, we wanted to create a more macrophage restricted Cre driver mouse line. To do this we established a novel knock-in mouse line hCD68-CreERT2, which expressed the tamoxifen inducible CreERT2 recombinase under the control of the human CD68 promoter and the regulatory element in IVS-1. We predicted that inducible Cre-recombinase activity would be more restricted to macrophages because it would avoid the early expression profile of CD68 during embryogenesis and haematopoiesis. Our goal was to overcome the limited macrophage specificity seen in available ‘macrophage specific’ targeting mouse lines.

## 2 Materials and Methods

### 2.1 Animal Studies

#### 2.1.1 Ethical Statement

All animal experiments were conducted in accordance to the Animal (Scientific Procedures) Act 1986, with procedures reviewed by the Sir William Dunn School of Pathology (SWDSOP) ethical review body (AWERB) and conducted under project **licence** P144E44F2 at the University of Oxford. Animals were housed in individually ventilated cages (between 2 and 6 mice per cage) in specific pathogen free conditions. All animals were provided with standard chow and water ad libitum and, maintained on a 12 h light: 12 h dark cycle at controlled temperature (20–22°C) and humidity. Breeding strategies were optimised to reduce numbers of animals used.

#### 2.1.2 Generation of hCD68-CreERT2 Mouse

A tamoxifen inducible Cre recombinase cDNA (CreERT2) was cloned downstream of a CD68 promoter, consisting of 2.9 kb of CD68 5’ flanking sequence with the 83-bp first intron (IVS-1) of the CD68 gene (PMID 11454064), together with a rabbit beta-globin polyadenylation sequence. This transgenic cassette was subcloned into the pExchange4-CB9 vector suitable for PhiC31 integrase mediated cassette exchange at the Gt(ROSA)26Sor locus (PMID 21853122). The resulting vector, CB9-CD68-CreERT2 contains a promoterless neomycin phosphotransferase cassette, upstream of CD68-CreERT2-pA and the whole array is flanked by PhiC31 attP sites.

CB9-CD68-CreERT2 DNA (5 µg) was electroporated into 1 × 106 RS-PhiC embryonic stem cells, a C57BL/6N JM8F6 derived line (PMID 26369329), using the Neon transfection system (Life Technologies) (3 × 1400 V, 10 ms), and selected in 210 µg/ml G418 for 7 days. Resistant colonies were isolated, expanded and screened for correct cassette exchange at the Gt(ROSA)26Sor locus using primers CAG-F (5′-CAGCCATTGCCTTTTATGGT-3) and ExNeo2 (5′-GTTGTGCCCAGTCATAGCCGAATAG-3′) to verify the 5’ integration event and att-F1 (5′-GCACTAGTTCTAGAGCGATCCCC-3’) and 3HR-R1 (5′-CGGGAGAAATGGATATGAAGTACTGGGC-3′) to verify the 3’ integration event. Recombinant ES cells were injected into albino C57BL/6J blastocysts, and the resulting chimeras were bred with albino C57BL/6J mice to confirm germline transmission.

#### 2.1.3 Generation of hCD68-CreERT2 LSL-tdTomato Mice

Gt(ROSA)26Sortm27.1(CAG-COP4*H134R/tdTomato) (LSL-tdTomato) mice were bought from The Jackson Laboratory and maintained in-house and genotype confirmed using PCR. LSL-tdTomato heterozygous females were crossed with hCD68-CreERT2 heterozygous males. Off-spring were then genotyped to confirm presence of transgenes. Mice which were heterozygous for both transgenes, hCD68-CreERT2 LSL-tdTomato (hCD68-tdTom) were used to assess Cre-recombinase efficacy in further experiments. Littermates were used as negative controls.

#### 2.1.4 Tamoxifen Preparation

Tamoxifen (catalog #156738; MP Biomedicals) was dissolved in 100% ethanol followed by dilution in peanut oil (catalog #P2144; Sigma‐Aldrich) at the final concentration of 10 mg/ml.

#### 2.1.5 Cre-Recombinase Induction Protocol

**Figure 1:**
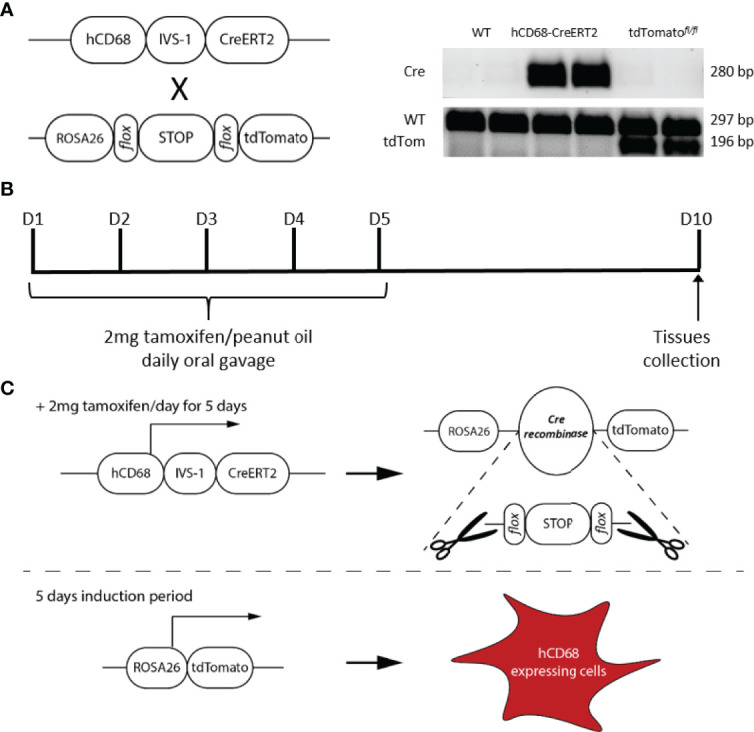
Design and generation of the inducible hCD68-CreERT2 model. **(A)** Presence of Cre recombinase and tdTomato genes was confirmed by extracting DNA from ear notches, a standard PCR amplification and running side-by-side on an agarose gel for all genotypes available for het x het breeding strategy (see methods for details). **(B)** Tamoxifen dosing protocol for induction of Cre recombinase activity in hCD68-CreERT2 model. **(C)** Principles of the human CD68 promoter-driven tamoxifen inducible Cre recombinase activation; not to scale.

8 to 13-week-old male and female mice were given tamoxifen (2 mg; for 5 days; *via* oral gavage) or peanut oil as vehicle control. Mice were killed humanely and tissues harvested 5 days or 6 weeks and 5 days after the final dose; refer to **Figures 1** and **5** for timeline.

### 2.2 Flow Cytometry

Cells were washed in FACS buffer (0.05% BSA, 2 mM EDTA in PBS, pH 7.4) blocked using anti-CD16/32 (1 µg; Biolegend) for 15 mins at 4°C, followed by 30 min antibody staining for the surface markers with isotype controls. Cells were again washed and resuspended in 200 µL of FACS buffer without fixation. Antibodies used are listed in **Table 1**, all bought from BioLegend apart from FITC-CD8a, which was bought from BD Biosciences.

**Table 1:** List of flow cytometry antibodies used in this study.

Marker	Fluorophore	Isotype	Clone
CD3	Brilliant Violet 510	Armenian Hamster IgG	145-2C11
CD4	PE/Cyanine7	Rat IgG2b	GK1.5
CD8a	FITC	Rat IgG2a	53-6.7
CD8a	PE	Rat IgG2a	53-6.7
CD11b	Brilliant Violet 421	Rat IgG2b	M1/70
CD11c	Brilliant Violet 650	Armenian Hamster IgG	N418
CD11c	PE/Cyanine7	Armenian Hamster IgG	N418
CD19	Pacific Blue	Rat IgG2a	6D5
CD45	APC/Cyanine7	Rat IgG2b	30-F11
CD45R/B220	APC	Rat IgG2a	RA3-6B2
CD68	PE	Rat IgG2a	FA-11
CD68	APC/Cyanine7	Rat IgG2a	FA-11
CD115	PE/Cyanine7	Rat IgG2a	AFS98
CD170 (Siglec-F)	APC	Rat IgG2a	S17007L
Ly-6C	FITC	Rat IgG2c	HK1.4
Ly-6C	PerCP/Cyanine5.5	Rat IgG2c	HK1.4
Ly-6C	PE	Rat IgG2c	HK1.4
Ly-6G	Brilliant Violet 510	Rat IgG2a	1A8
F4/80	PE	Rat IgG2a	BM8
F4/80	APC	Rat IgG2a	BM8
F4/80	FITC	Rat IgG2a	BM8
F4/80	APC/Cyanine7	Rat IgG2a	BM8
I-A/I-E (MHCII)	FITC	Rat IgG2b	M5/114.15.2
I-A/I-E (MHCII)	PE	Rat IgG2b	M5/114.15.2
CLEC4F	Alexa Fluor 647	Mouse IgG1	3E3F9
Tim-4	PE/Cyanine7	Rat IgG2a	RMT4-54

Intracellular staining was performed as per manufacturer’s instructions using eBioscience FOXP3/Transcription Factor Staining Buffer Set (invitrogen). Briefly, after the last wash of the extracellular staining protocol, cells were fixed in Fixation/Permeabilization Buffer for 30 min at room temperature. Cells were washed and resuspended in Permeabilization Buffer; intracellular antibodies were added for 30 min in room temperature. Cells were again washed in Permeabilization Buffer and resuspended in FACS buffer for flow cytometric analysis.

Data were acquired using BD Fortessa X20 cytometer and Diva software (BD Biosciences) and then analysed using FlowJo (Tree Star Inc, USA) software. Fluorescence minus One (FMO) controls were used for gating and single stains for compensation were obtained using UltraComp eBeads™ Compensation Beads (InVitrogen) for liver, spleen and blood panels and single stained cell samples for peritoneal cells.

### 2.3 Fluorescent Microscopy

Mice were perfused with 10-20 mL of cold PBS and organs were harvested into 4% paraformaldehyde and incubated at 4oC for 6 hours before transferring into 30% sucrose overnight to dehydrate. Samples were then embedded OCT matrix and sectioned at –20oC at 5 µm per section. Sections were then countered-stained with DAPI. Images were taken using Live Cell Olympus Confocal Microscope.

### 2.4 Hyperlipidaemia Model

hCD68-tdTom male and female mice and their control littermates were injected intravenously once with AAVmPCSK9 (AAV.8TBGmPCSK9D377Y, Penn Vector Core) at 5×1011 viral particles/mouse and placed onto a high fat and cholesterol diet (SDS 829108 Western RD diet) for 16 weeks. After 16 weeks mice were orally gavaged with 2 mg of tamoxifen or peanut oil as a vehicle control for 5 consecutive days and tissues were collected 5 days later; refer to **Figure 7** for timeline.

### 2.5 Oil Red O Staining

Perfusion fixed frozen hearts were embedded in OCT and cut in 5 μm sections. Sections were brought to room temperature, washed with 60% isopropanol, then saturated with Oil Red O (1% w/v, 60% isopropanol) for 15 min, washed in 60% isopropanol and rinsed in distilled water. Sections were then counterstained with haematoxylin. Coverslips were applied in aqueous mounting medium.

### 2.6 Aortic Digestion

All enzymes were purchased from Sigma-Aldrich, unless stated otherwise. Tissues were isolated in 5% FBS in RPMI-1640 (Gibco). Tissues were cut into small pieces and digested in the enzyme cocktail (450 U/ml collagenase I, 125 U/ml collagenase XI 60 U/ml hyaluronidase and 60 U/ml DNase I (ThermoFisher Scientific)) at 37 °C in shaker for 50 min. The cells were retrieved by passing tissue pieces through a 70 μm cell strainer (Greiner Bio-One) and processed for immunofluorescent staining for flow cytometry (31).

### 2.7 Statistical Analysis

All experiments were designed, where possible, to generate groups of equal size. Where possible, blinding and randomisation protocols were used. All data in the text and figures are presented as mean ± standard error mean (SEM) of n observations, where n represents the number of animals studied (*in vivo*) or independent values, not technical replicates (*in vitro*). All statistical analysis was calculated using GraphPad Prism 9 (GraphPad Software, San Diego, California, USA; RRID : SCR_002798). When the mean of two experimental groups were compared, a two-tailed Students t-test was performed. Normally distributed data without repeated measurements were assessed by a one-way ANOVA followed by Bonferroni correction if the F value reached significance; two-way ANOVA was used for comparing control and experimental mice across two separate conditions In all cases a P < 0.05 was considered significant.

## 3 Results

### 3.1 Generation of hCD68-CreERT2-tdTomato Reporter Mouse

To exploit the macrophage targeting activity of the human CD68 promoter which includes the regulatory element IVS-1 (28, 32) we engineered a DNA construct that encoded a tamoxifen inducible Cre recombinase (33) under the hCD68 promoter regulatory region, this was then inserted into the ROSA26 locus of embryonic stem cells before being injected into albino C57BL/6J blastocysts yielding hCD68-CreERT2 chimeric founders. Mice were fertile and produced an expected Mendelian ratio of transgenic and non-transgenic litter mates with no obvious developmental defects. Genotype was confirmed by PCR. Heterozygous offspring were then bred with LSL-tdTomato mice to generate double transgenic experimental mice (hCD68-tdTom) (**Figure 1A**), littermates carrying no transgenes, or a single transgene were used as controls; out of 444 pups born during this study 109 were heterozygous for both alleles, 101 mice were heterozygous for hCD68-CreERT2 only, 102 mice were heterozygous for tdTomato only and 132 mice were wild-typed for both alleles. The optimal Cre recombinase induction protocol was determined to be administration of tamoxifen (2 mg per mouse; p.o.) for 5 days, and collection of cells and tissues 5 days later (**Figure 1B**).

### 3.2 The hCD68 Promotor Drives Inducible Cre-Recombinase in Tissue Resident Macrophages

**Figure 2:**
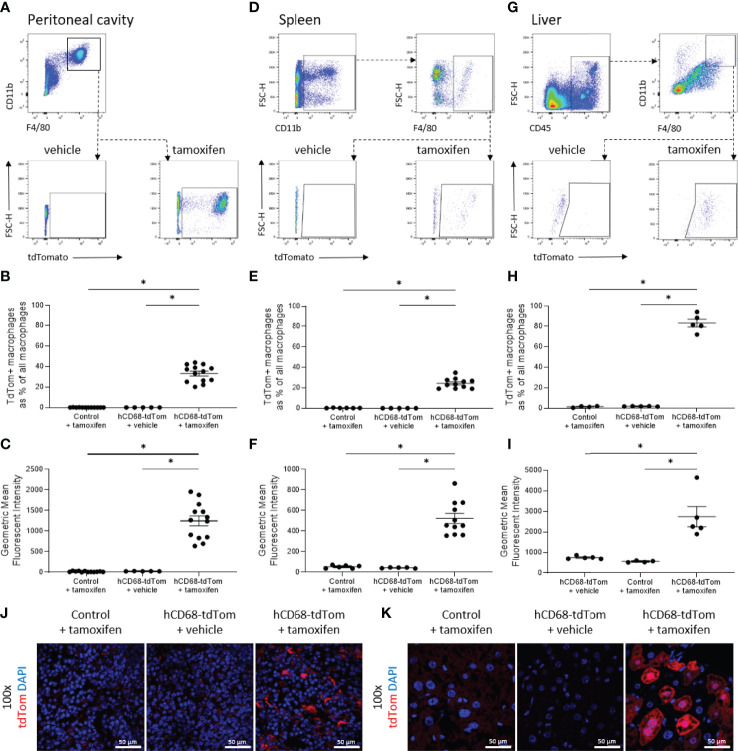
The hCD68 promoter driven inducible Cre recombinase has high activity levels in tissue resident macrophages. Tissue-resident macrophage populations in **(A)** peritoneal cavity, **(D)** spleen and **(G)** liver were defined as CD11b^+^ and F4/80^+^ cells (gating strategy shown by representative dot plots) with the corresponding frequency of tdTomato-expressing macrophages as a proportion of all macrophages shown in **(B)** peritoneal cavity, **(E)** spleen and **(H)** liver and with respective mean fluorescence intensity values of tdTomato expression in the whole macrophage population in **(C)**, **(F)** and **(I)**; one-way ANOVA (* = p < 0.05). Representative immunofluorescence images of endogenous tdTomato expression in **(J)** the spleen and **(K)** the liver at 100x magnification with DAPI in blue and tdTomato in red; scale bar = 50µm.

In order to assess the pattern of inducible Cre activity in tamoxifen treated hCD68-tdTom mice in different tissue resident macrophage populations we used flow cytometry with panels of well characterised monoclonal antibodies. When compared to vehicle treated hCD68-tdTom mice, tamoxifen treated hCD68-tdTom mice had robust expression of tdTomato in CD11b^+^F4/80^+^ peritoneal resident macrophages (**Figures 2A–C**). We further investigated the level of tdTomato expression in two known peritoneal macrophage populations (**Supplementary Figure 1**). When compared to vehicle treated hCD68-tdTom mice, in tamoxifen treated hCD68-tdTom mice ~40% of LPM displayed tdTomato expression, while ~ 20% of SPM displayed tdTomato expression, moreover, the level of tdTomato expression (MFI) was significantly lower in SPM compared to LPM.

Within the spleen of tamoxifen treated hCD68-tdTom mice there was robust expression of tdTomato in CD11b^+^F4/80^+^ macrophages (**Figures 2D–F**). As the spleen is a reservoir for multiple myeloid cell types tdTomato expression was quantified in other myeloid cell populations within the spleen. Monocytes were sub-divided into Ly6C^hi^ (CD11b^+^CD115^+^Ly6C^hi^) and Ly6C^low^ (CD11b^+^CD115^+^Ly6C^low^) (**Supplementary Figure 2A**). Importantly, <5% of Ly6C^hi^ monocytes had tdTomato expression (**Supplementary Figure 2B**) while ~27% of the splenic Ly6C^low^ monocytes expressed tdTomato (**Supplementary Figure 2C**). On the other hand, < 4% of neutrophils (CD11b^+^Cd115^-^Ly6G^+^) expressed tdTomato (**Supplementary Figure 2D**). We also found that ~20% of splenic dendritic cells (CD45^+^CD11c^+^MHCII^+^) had tdTomato expression (**Supplementary Figures 2E/F**). In the non-myeloid cells ~ 2.5% of splenic T-cells (CD45^+^CD11b^-^CD3^+^) (**Supplementary Figure 2E/G**) and 5% B-cells (CD45^+^CD11b^-^B220^+^) expressed tdTomato (**Supplementary Figure 2E/H**).

The liver has a large and heterogenous population of tissue resident macrophages (34). When compared to vehicle-treated hCD68-tdTom mice, tamoxifen treated hCD68-tdTom mice had robust expression of tdTomato in all liver macrophage populations. Within the liver ~80% of CD11b^hi^F4/80^hi^ macrophages from tamoxifen treated hCD68-tdTom mice expressed high levels of tdTomato (**Figure 2G-I**). However, within the liver there is significant heterogeneity within the macrophage population (**Supplementary Figure 3**); we therefore wanted to further investigate hCD68 promoter driven Cre activity within different macrophage subsets. The populations assessed were lipid-associated macrophages (LAMs; CD11b^hi^Ly6C^+^CD11c^-^MHCII^+^CLEC4F^low^; **Supplementary Figure 3B**), monocyte-derived Kupffer cells (Mono-KC; CD11b^hi^Ly6C^+^CD11c^-^MHCII^+^CLEC4F^+^; **Supplementary Figure 2C**), capsular macrophages (CM; CD11b^low^F4/80^int^TIM4^-^MHCII^+^; **Supplementary Figure 3D**); Kupffer cells (KC; CD11b^+^F4/80^hi^CLEC4F^hi^TIM4^hi^; **Supplementary Figure 3E**). All liver macrophage populations in tamoxifen treated hCD68-tdTom mice expressed tdTomato, however the intensity of tdTomato was different between subsets of liver macrophage (**Supplementary Figure 3F**). Mono-KCs expressed the highest levels of tdTomato, while CMs expressed the lowest level (**Supplementary Figure 3F**).

To confirm flow cytometry results we used fluorescent microscopy to visualize endogenous tdTomato expression in the spleen and liver. Control spleens (**Figure 2J**) and liver (**Figure 2K**) displayed no expression of tdTomato. However, tamoxifen treated hCD68-tdTom mice displayed a discrete expression pattern of tdTomato within the spleens (**Figure 2J**) and liver (**Figure 2K**) that was consistent with data obtained using flow cytometry.

### 3.3 The hCD68 Promotor Drives Limited Inducible Cre-Recombinase Expression in Circulating Leukocytes

**Figure 3:**
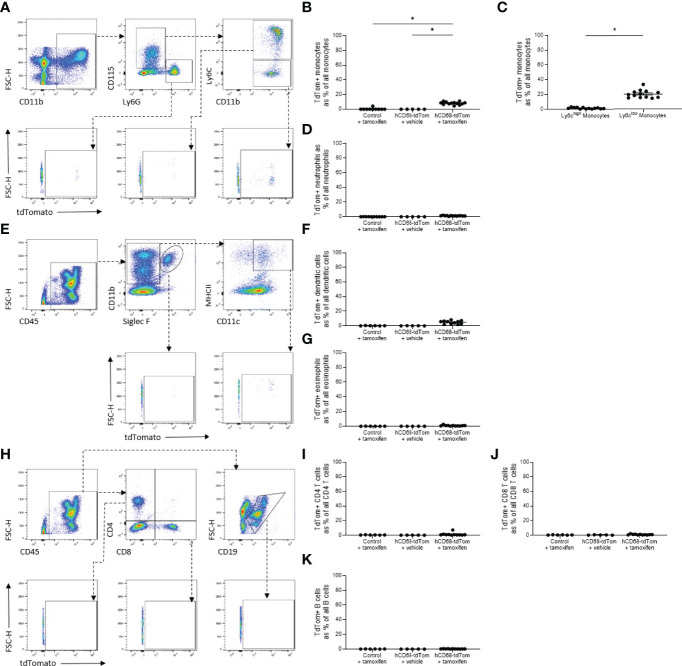
The hCD68 promoter driven inducible Cre recombinase has limited activity in circulating leukocytes. **(A)** Representative dot plots of the gating strategy for neutrophils (CD11b^+^, CD115^-^, Ly6G^+^), total monocytes (CD11b^+^, CD115^+^, Ly6G^-^), Ly6C^high^ and Ly6C^low^ monocytes (CD11b^+^, CD115^+^, Ly6G^-^, Ly6C^+^ and CD11b^+^, CD115^+^, Ly6G^-^, Ly6C^-^ respectively). **B)** Frequency of tdTomato-expressing monocytes as a proportion of all monocytes. **(C)** Frequency of tdTomato-expressing Ly6C^high^ and Ly6C^low^ monocytes. **(D)** Frequency of tdTomato-expressing neutrophils as a proportion of all neutrophils. **(E)** Representative dot plots of the gating strategy for dendritic cells (CD45^+^, CD11b^+^, Siglec F^-^, MHCII^+^, CD11c^+^) and eosinophils (CD45^+^, CD11b^+^, Siglec F^+^). **(F)** Frequency of tdTomato-expressing dendritic cells as a proportion of all dendritic cells. **(G)** Frequency of tdTomato-expressing eosinophils as a proportion of all eosinophils. **(H)** Representative dot plots of the gating strategy for CD4 T cells (CD45^+^, CD4^+^, CD8^-^), CD8 T cells (CD45^+^, CD4^-^, CD8^+^) and B cells (CD45^+^, CD19^+^). **(I)** Frequency of tdTomato-expressing CD4 T cells as a proportion of all CD4 T cells. **(J)** Frequency of tdTomato-expressing CD8 T cells as a proportion of all CD8 T cells. **(K)** Frequency of tdTomato-expressing B cells as a proportion of all B cells; one-way ANOVA or student t-test as appropriate, (* = p < 0.05).

Previously the hCD68 protomer was used to drive green fluorescent protein and was shown to have robust expression profiles in non-macrophage myeloid cell populations namely, monocytes and neutrophils (29, 30). Therefore, Cre-recombinase activity was assessed in circulating myeloid and non-myeloid leukocyte populations. There was no detectable tdTomato expression in circulating CD11b^+^CD115^+^ monocytes from control mice, however, tamoxifen treated hCD68-tdTom mice had a small but significant increase in the number of tdTomato expressing cells (**Figure 3B**). We further assessed the level of tdTomato expression in Ly6C^high^ and Ly6C^low^ monocytes. Interestingly, we only saw evidence of td-Tomato expression in Ly6C^low^ monocytes, suggesting that the inducible hCD68 promotor driven Cre-recombinase has activity in patrolling Ly6C^low^ monocytes (**Figure 3C**). Other myeloid Cre driver lines have significant activity in neutrophils. We therefore wanted to ascertain if the inducible hCD68 driven Cre recombinase had any activity in circulating neutrophils. Tamoxifen treated hCD68-tdTom mice had no or very low expression of tdTomato (<2%) in CD11b^+^CD115^-^Ly6G^+^ neutrophils (**Figure 3D**). Similarly, there was <5% expression of tdTomato in dendritic cells from tamoxifen treated hCD68-tdTom mice and <1% tdTomato expression in eosinophils from tamoxifen treated hCD68-tdTom mice (**Figures 3F, G** respectively). Additionally, there was almost no tdTomato expression in CD4^+^, CD8^+^ T-cells or in circulating B-cells in tamoxifen treated hCD68-tdTom mice (**Figures 3I-K** respectively). Taken together, these results demonstrate that hCD68 driven inducible Cre recombinase is highly selective for macrophage populations *in vivo* and has little activity in circulating myeloid and lymphoid cell populations.

### 3.4 The Human CD68 Promoter Has Higher Macrophage Specificity Compared to Endogenous Murine Cd68 Expression

**Figure 4:**
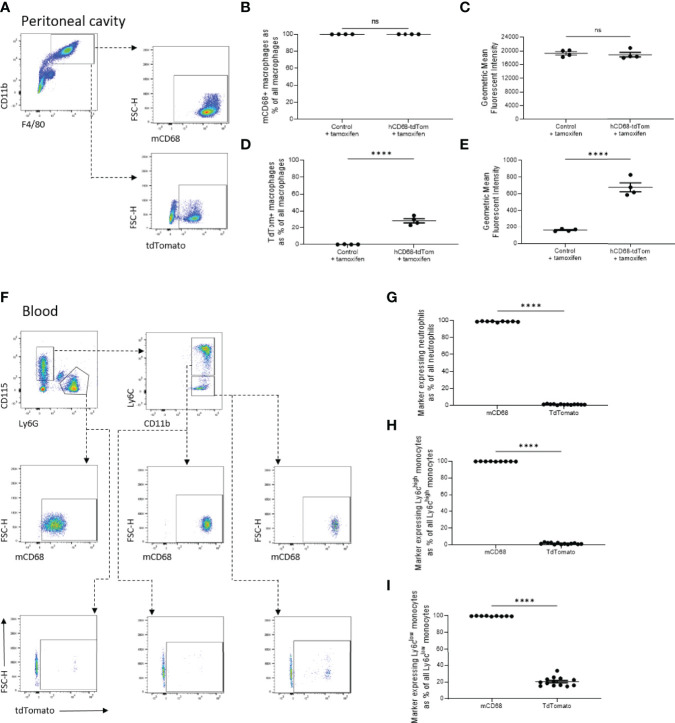
The transgenic human CD68 promoter has higher macrophage specificity compared to endogenous murine Cd68 expression. **(A)** Representative dot plots of the gating strategy for peritoneal macrophages (CD11b^+^, F4/80^+^) and their expression of tdTomato and murine CD68 (mCD68). **(B)** Frequency of tdTomato-expressing peritoneal macrophages as a proportion of all peritoneal macrophages with corresponding mean fluorescence intensity in **(C)**. **(D)** Frequency of mCD68-expressing macrophages as a proportion of all peritoneal macrophages with corresponding mean fluorescence intensity in **(E)**. **(F)** Representative dot plots of the gating strategy for blood neutrophils (CD115^-^, Ly6G^+^), Ly6C^high^ and Ly6C^low^ monocytes (CD115^+^, Ly6G^-^, Ly6C^+^ and CD115^+^, Ly6G^-^, Ly6C^-^ respectively) and their expression of tdTomato and mCD68. **(G)** Comparison of frequency of neutrophils expressing mCD68 and tdTomato. **(H)** Comparison of frequency of Ly6C^high^ monocytes expressing mCD68 and tdTomato. **(I)** Comparison of frequency of Ly6C^low^ monocytes expressing mCD68 and tdTomato; for panels **(G–I)** mCD68 was quantified for control mice without tamoxifen, tdTom was measured for hCD68-tdTom + tamoxifen mice, student t-test (**** = p < 0.0001). ns, not significant.

Having shown that hCD68 driven inducible Cre recombinase has selective activity in macrophage populations, versus other myeloid cell populations, we postulated that this could mirror differing levels of the murine Cd68 expression. Even though all CD11b^+^F4/80^+^ tissue resident peritoneal macrophages express murine Cd68 (**Figures 4C**), only around ~30% of the same population express tdTomato (**Figure 4D/E**). In the circulation (**Figure 4F**), murine Cd68 was highly expressed by all neutrophils (CD11b^+^CD115^-^Ly6G^+^) (**Figure 4G**), Ly6C^high^ (**Figure 4H**) and Ly6C^low^ (**Figure 4I**) monocytes, but they expressed low to undetectable levels of tdTomato. These results show that the human CD68 promoter has increased lineage specificity for macrophages compared to expression of the murine Cd68/macrosialin.

### 3.5 TdTomato Expression in Tissue Resident Macrophages Is Maintained for at Least 6 Weeks After Cre Recombinase Activation

**Figure 5:**
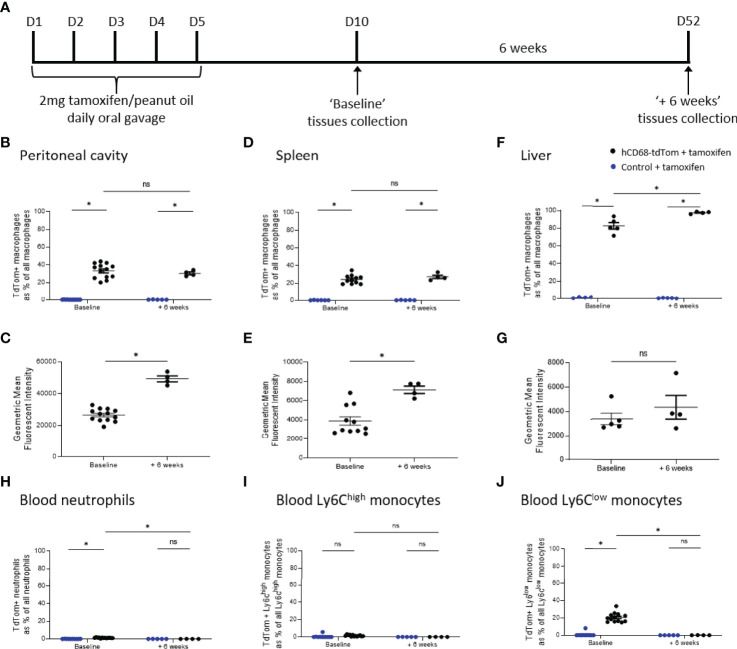
TdTomato expression in tissue resident macrophages is maintained for at least 6 weeks after Cre recombinase activation. In panels **(B, D, F, H–J)** control + tamoxifen mice are marked as blue dots; hCD68-tdTom + tamoxifen mice are marked as black dots. In all panels mice with the usual 5 days wash out period are marked ‘Baseline’, while mice with an additional 6 weeks wait are marked ‘+ 6 weeks’. **(A)** Tamoxifen dosing protocol for induction of Cre recombinase activity and assessment of targeting efficiency 6 weeks post-induction in hCD68-CreERT2 model. Frequency of tdTomato-expressing macrophages as a proportion of all macrophages in **(B)** peritoneal cavity **(D)** spleen and **(F)** liver with corresponding mean fluorescence intensity of tdTomato^+^ macrophages for ‘Baseline’ and ‘+6 weeks’ groups in **(C, E, G)**. Frequency of tdTomato-expressing blood **(H)** neutrophils, **(I)** Ly6C^high^ monocytes and **(J)** Ly6C^low^ monocytes as a proportion of all blood leukocytes of the given type for ‘Baseline’ and ‘+6 weeks’ groups; two-way ANOVA in panels **(B, D, F, H–J)**, student t-test in panels **(C, E, G)**, (* = p < 0.05). ns, not significant.

To test the longevity of tdTom expression following hCD68 driven Cre recombinase induction a 6-week protocol was used; baseline tdTom expression was determined 5 days after the last tamoxifen dose and a second group of mice (+ 6 weeks) was harvested after 6 weeks later (**Figure 5A**). tdTomato expression was assessed in macrophage populations within the peritoneal cavity, spleen and liver macrophages and in circulating leukocytes at baseline and + 6 weeks. There was no decrease in the number of tdTomato expressing macrophages in the peritoneal cavity (**Figure 5B**), spleen (**Figure 5D**) and liver (**Figure 5F**) in the +6 weeks group, however, there was increase in the intensity of tdTomato expression in the peritoneal cavity (**Figure 5C**) and spleen (**Figure 5E**) compared to baseline. Within circulating immune cell populations there was no expression of tdTomato in circulating neutrophils (**Figure 5H**) or Ly6C^hi^ monocytes (**Figure 5I**) and in the +6 weeks group and there was a significant decrease in the number of tdTomato expressing Ly6C^low^ monocytes (**Figure 5J**) compared to baseline.

### 3.6 The Human CD68 Promotor-Driven Inducible Cre Recombinase Improves Targeting of Liver Macrophages Compared to LysM-Cre.

**Figure 6:**
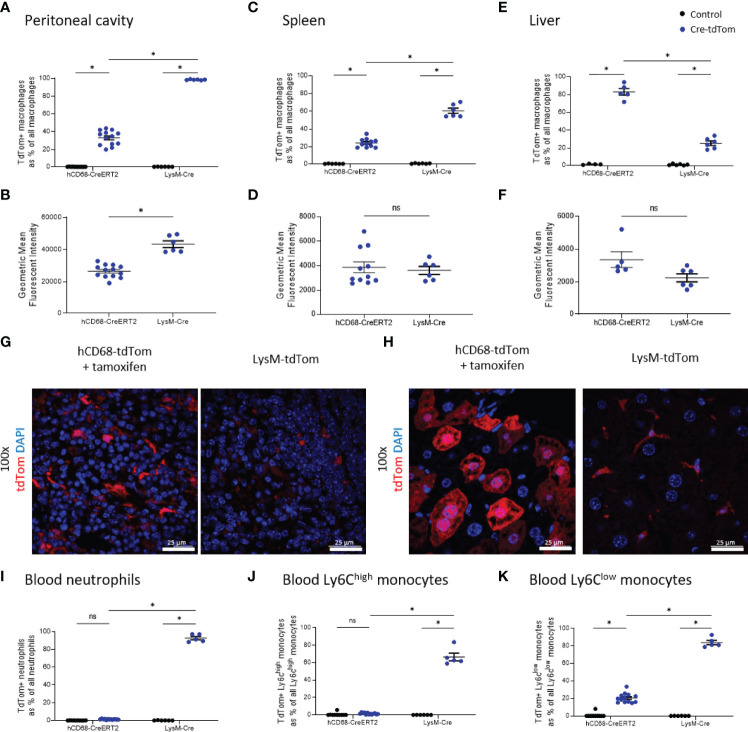
The hCD68 promoter driven inducible Cre recombinase improves targeting of liver macrophages compared to LysM-Cre. In all panels control mice (littermates of hCD68-tdTom mice that were given tamoxifen or littermates of LysM-tdTom mice) are marked as black dots; Cre-tdTom mice (hCD68-tdTom + tamoxifen or LysM-tdTom mice) are marked as blue dots. Frequency of tdTomato-expressing macrophages as a proportion of all macrophages in **(A)** peritoneal cavity **(C)** spleen and **(E)** liver with corresponding mean fluorescence intensity of tdTomato^+^ macrophages for hCD68-CreERT2 and LysM-Cre mice in **(B, D, F)**. Representative immunofluorescence images of endogenous tdTomato expression in **(G)** the spleen and **(H)** the liver of hCD68-tdTom and LysM-tdTom mice at 100x magnification with DAPI in blue and tdTomato in red; scale bar = 25 µm. Frequency of tdTomato-expressing blood **(I)** neutrophils, **(J)** Ly6C^high^ monocytes and **(K)** Ly6C^low^ monocytes as a proportion of all blood leukocytes of the given type for hCD68-CreERT2 and LysM-Cre mice.; two-way ANOVA in panels **(A, C, E, I–K)**, student t-test in panels **(B, D, F)**, (* = p < 0.05). ns, not significant.

The LysM-Cre is one of the most widely used macrophage Cre driver mice. We therefore, wanted to compare tdTomato expression in tamoxifen treated hCD68-tdTom mice with constitutively activated LyM-Cre-tdTom mice. Cre activation in both hCD68-tdTom and LysM-Cre-tdTom resulted in tdTomato expression in macrophages in the peritoneal cavity (**Figure 6A/B**), spleen (**Figure 6C/D**) and liver (**Figure 6E/F**). Peritoneal cavity macrophages from LysM-Cre-tdTom mice, also had higher levels of tdTom expression (**Figure 6B**), while no difference in MFI was seen in splenic macrophages (**Figure 6D**). Livers from tamoxifen treated hCD68-tdTom had significantly more tdTomato expressing macrophages than livers from LysM-Cre-tdTom mice (**Figure 6E**), however, there was no difference in MFI (**Figure 6F**). Fluorescent microscopy to visualize tdTomato expression and confirmed flow cytometry results in the spleen (**Figure 6G**) and liver (**Figure 6H**).

We next wanted to examine the specificity of the hCD68-CreERT2 expression by assessing tdTomato in circulating leukocyte populations. We demonstrated that tamoxifen treated hCD68-tdTom mice have almost no expression of tdTomato on blood neutrophils (**Figure 6I**); however, ~90% of circulating neutrophils in LysM-Cre-tdTom mice expressed tdTom (**Figure 6I**). A similar result was seen in blood Ly6C^hi^ monocytes; only ~2.5% of Ly6C^hi^ monocytes in tamoxifen treated hCD68-tdTom expressed tdTomato, whereas ~66% of Ly6C^hi^ monocytes from LysM-Cre-tdTom mice expressed tdTomato (**Figure 6J**). Additionally, ~20% of Ly6C^low^ monocytes from tamoxifen treated hCD68-tdTom expressed tdTomato, whereas ~83% of Ly6C^low^ monocytes from LysM-Cre-tdTom mice expressed tdTomato (**Figure 6K**).

### 3.7 The Human CD68 Promotor-Driven Inducible Cre Recombinase Targets Recruited Macrophages in Atherosclerotic Lesions

**Figure 7:**
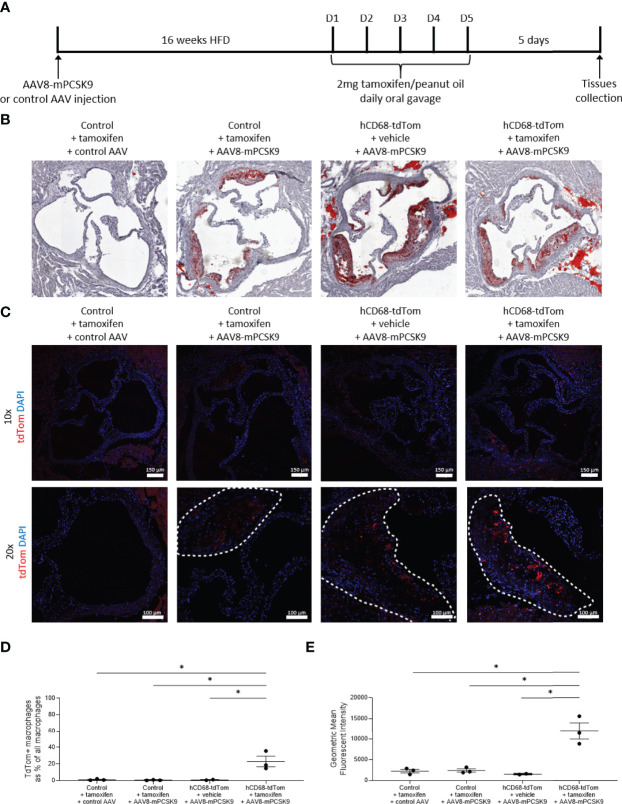
The hCD68 promoter driven inducible Cre recombinase targets recruited macrophages in the atherosclerotic lesion. **(A)** Timeline of the inducible PCSK9 atherosclerosis model and induction of Cre recombinase in hCD68-CreERT2 mice. **(B)** Representative images of Oil Red O staining of the aortic roots confirm the induction of atherosclerotic plaques in the AAV8-mPCSK9 injected groups. **(C)** Representative immunofluorescence images of endogenous tdTomato expression in the aortic roots at 10x (whole aortic root) and 20x (plaque close-up with plaque edges outlined with the dashed line) magnification with DAPI in blue and tdTomato in red; scale bar = 150 µm in 10x images and 100 µm in 20x images. **(D)** Frequency of tdTomato-expressing aortic macrophages as a proportion of all aortic macrophages with the corresponding mean fluorescent intensity of total aortic macrophages in **(E)**; one-way ANOVA (* = p < 0.05).

Lipid laden macrophages within arterial walls are a key driver of the pathophysiology in cardiovascular disease. To induce hyperlipidaemia hCD68-tdTom and control mice were injected with a gain-of-function mutant PCSK9 adeno-associated virus vector (AAV) or a control viral vector (35). Mice given a single intravenous injection of AAV8-mPCSK9 or control AAV were placed on a high fat diet (HFD). After 16 weeks of HFD feeding hCD68-tdTom mice or control were given tamoxifen to induce Cre recombinase activity (**Figure 7A**). As expected, mice given a single injection of AAV8-mPCSK9 but not a control AAV developed atherosclerotic lesions in the aortic sinus as shown by increased Oil Red O staining (**Figure 7B**). We next looked for tdTomato expression within atherosclerotic lesions in tamoxifen treated hCD68-tdTom mice. AAV8-mPCSK9 injected hCD68-tdTom mice treated with tamoxifen expressed high levels of tdTomato within the atherosclerotic plaque. (**Figure 7C**). TdTomato expressing macrophages were distinguishable from auto fluorescent lipid drops, due to the proximal co-localisation of DAPI stained nuclei (**Supplementary Figure 4A**).

To further assess the tdTom expression in the immune cells compartment of the atherosclerotic lesion we preformed aortic digests followed by flow cytometry (**Supplementary Figure 4B**). As expected, there was a large population of CD11b^+^F4/80^+^CD64^+^ macrophages in AAV8-mPCSK9 injected mice. Importantly, when these mice were treated with tamoxifen to induce Cre recombinase activity there was significant induction in expression of tdTomato (**Figure 7D**). We observed tdTomato expression in ~25% of all Ly6C^+^ monocyte derived macrophages within in the atherosclerotic lesion (**Supplementary Figure 4C**), and reassuringly there was no tdTomato expression in recruited neutrophils (**Supplementary Figure 4D**). As expected within the blood (**Supplementary Table 1**) and the spleen (**Supplementary Table 2**) there was limited td-tomato expression in all major leukocyte populations except Ly6C^low^ monocytes in AAV8-mPCSK9 injected hCD68-tdTom mice treated with tamoxifen.

Of note when compared to baseline, AAV8-mPCSK9 injected hCD68-tdTom mice treated with tamoxifen and fed a HFD for 16 weeks did not have different levels of tdTomato expression in the peritoneal cavity (**Supplementary Figure 5A/B**) or spleen macrophages (**Supplementary Figure 5C/D**). Additionally, as expected, in the blood there was no difference in td-Tomato expression in neutrophils (**Supplementary Figure 5E/F**), Ly6C^high^ (**Supplementary Figure 5G/H**) and Ly6C^lo^ monocytes (**Supplementary 5Figure I/J**). Of note, there was a higher number of td-Tomato expressing Ly6C^hi^ monocytes within the spleen of atherogenic mice (4.25 ± 0.54 vs 13.50 ± 2.69; **Supplementary Table 3**). Collectively, these results demonstrate that the human CD68 promoter-driven inducible Cre recombinase can specifically target macrophages with atherosclerotic lesions, with limited targeting to plaque associated neutrophils.

## 4 Discussion

We have generated and characterised a novel transgenic reporter mouse expressing tamoxifen inducible Cre recombinase under the control of the human CD68 promoter. To assess the efficiency of Cre recombinase activation we crossed the hCD68-CreERT2 mice with commercially available TdTomato^flox/flox^ mice, a standard way of phenotyping new transgenic Cre driver lines. TdTomato-expressing cells were detected using flow cytometry and confocal microscopy in resident macrophage populations in the peritoneal cavity, spleen and liver (**Figure 2**). Detailed analysis showed that inducible tdTomato expression is restricted to tissue-resident macrophages, absent from the circulating leukocytes and independent from murine Cd68 expression (**Figures 3** and **4** respectively). We tested the longevity of Cre recombinase targeting by allowing 6 weeks wash-out period following the usual dosing protocol and showed no decline in tdTomato expression in tissue-resident macrophage populations (**Figure 5**). Additionally, we performed a side-by-side comparison with LysM-Cre mice (using the same tdTomato^flox/flox^ mouse colony for breeding) and showed better macrophage lineage-restricted expression of tdTomato in our model (**Figure 6**). As a proof-of-concept experiment, we induced atherosclerotic lesions in hCD68-tdTom mice using the PCSK9 model (35) and showed that it is possible to target macrophages recruited to the plaques with our model (**Figure 7**). Taken together, our experiments show that hCD68-CreERT2 mouse line allows efficient and specific targeting of tissue-resident macrophage populations and can be used in long term studies and disease models.

The search for a truly ‘macrophage-specific’ transgene targeting has so far been unsuccessful, most likely due to all myeloid cells expressing very similar markers at various stages of development (36, 37). CD68 is a classical macrophage-defining marker, frequently used to identify macrophages in histological sections (38). In our new mouse line, tdTomato expression in circulating leukocytes of myeloid origins (neutrophils, monocytes and dendritic cells) is extremely low (**Figure 3**). This is a crucial difference to our earlier work, where we used the hCD68 promoter IVS-1 cassette and saw constitutive expression of green fluorescent protein in neutrophils, monocytes and macrophages alike (29). We hypothesised that the difference in transgene expression might result from differential expression of murine Cd68 across the myeloid populations in the blood. Contrary to our hypothesis, we saw that macrosialin was highly expressed in all myeloid populations in the peritoneum and blood, including the neutrophils (**Figure 4**). It has been previously reported that monocytes and a subset of human blood neutrophils can express CD68, but not to the extent we saw in our study (39). The human CD68 promoter and IVS-1 intron expression cassette has been shown to be a very potent driver for macrophage targeting when used in a constitutive GFP transgenic mouse (29). However, due to the nature of this cassette, when activated embryonically it targets other myeloid cell types with efficiency comparable to macrophage targeting, in line with Cd68 being expressed by other myeloid cell types. Iqbal *et al.* demonstrated that hCD68-GFP protein was detected as early as day E8.5, which is before distinct hematopoietic progeny are present and the emergence of *bone fide* macrophages within the hematogenic endothelium between E10.5 and E12.5. Similar results have been seen in other inducible hCD68 promoter-driven mice models, where reported expression in blood leukocytes was lower than in other macrophage targeting Cre lines (13, 40). This suggests that human CD68 promoter cassette when activated in adult animals is highly specific for mature tissue resident macrophages, unlike the endogenous expression pattern of murine Cd68.

The main limitation of our transgenic model lies in the fact that we do not achieve full targeting in all tissue resident macrophage populations. This is however unsurprising given that none of the previously published macrophage targeting inducible Cre models achieved full expression in desired cell populations (13, 40, 41). We achieved the highest efficiency of targeting in the liver, which might be due to tamoxifen being metabolised in the liver to its active form (4-hydroxy tamoxifen) and therefore having the highest bioavailability in that tissue (42). Our mouse line achieved better expression in liver macrophages in comparison to LysM Cre mice, which are the gold standard of macrophage targeting lines (**Figure 6**). Moreover, we were able to discriminate between different sub-populations of liver macrophages and show that Cre recombinase activation varies between them in terms of efficiency of targeting as well as intensity of expression (**Supplementary Figure 3**). Kupffer cells were the highest expressors of tdTomato in the liver with much lower values in the monocyte-derived sub-populations and in capsular macrophages, reinforcing the idea that hCD68-CreERT2 mouse line preferentially targets tissue resident macrophages. Moreover, we were able to distinguish between macrophage subsets based on significant differences in their mean fluorescence intensity values (34). Encouragingly, we saw no decline in tdTomato expression 6 weeks post-targeting (**Figure 5**), which further points to the targeting of tissue-resident macrophages, which are known to have a slow turnover (43). In contrast, after the 6 weeks wash-out period there was no expression of tdTomato in the blood at all, since circulating blood cells tend to be short-lived (44).

In a proof of concept experiment we were able to target recruited monocytes and macrophages in a widely used animal model of atherosclerosis. We were able to show that aortic-resident and recruited monocyte-derived macrophages expressed tdTomato, proving the possibility of using our system to study macrophage biology in pathological states (**Figure 7** and **Supplementary Figure 4**). The relatively high level of monocyte targeting compared to homeostatic state in other tissues and in blood could be because monocytes that have migrated into the aortic tissue have most likely started the phenotypic shift towards macrophages and therefore upregulated the expression of hCD68 promoter cassette.

Cre driver mouse lines which are referred to as macrophage-targeting are driven by five main gene expression cassettes, LysM, Csf1r, Cx3cr1, CD11b and F4/80, however none of them are truly macrophage-specific. LysM-Cre was shown by us and others to target all myeloid populations, whilst having relatively low levels of expression in tissue-resident macrophages in spleen and liver (45). Csf1r-Cre mouse was noted to target at least 50% of neutrophils and T cells alongside bone-marrow derived macrophages, while other fluorescent reporter mice driven by Csf1r gene were shown to have high levels of targeting in megakaryocytes and dendritic cells (41, 46). Cx3cr1 promoter cassette has been used to create both constitutive and tamoxifen-inducible Cre mouse lines, however in comparative analysis with other macrophage targeting Cre lines showed that Cx3cr1-Cre had relatively low efficiency of targeting tissue-resident macrophages in the spleen and the lungs whilst having significant spill-over expression into mast cells and dendritic cells (8, 47). CD11b is a pan-myeloid marker and therefore, as expected, induced very high expression in all cells of myeloid lineage, while F4/80 was shown to target only selected tissue-resident macrophage populations due to differences the level of expression of F4/80 between tissues, making both of these mice a fairly rare choice for studying tissue resident macrophages (48, 49).

As we are gaining a better understanding of tissue-resident macrophages, their origins and ways to replenish them, it is crucial to develop more macrophage specific models that can be used for *in vivo* investigations. So far, the most detailed descriptions of heterogeneous macrophage populations have come from single cell studies, which have their own limitations and constraints in terms of availability to researchers and experimental design. Currently available macrophage-targeting mouse models offer little reassurance that circulating monocytes and neutrophils will not be equally well targeted. In this study we present a novel human CD68 promoter cassette driven inducible CreERT2 mouse model with the potential to improve tissue resident macrophage targeting *in vivo*. At the steady state, we showed improved specificity of targeting macrophages and minimal off-target effects in the blood, compared to other published mouse Cre driver lines. Moreover, we showed stable targeting over long periods of time and that it is possible to induce the Cre recombinase expression in disease models without disrupting the model itself. We believe that the hCD68-CreERT2 mice will find multiple applications in studying tissue-resident macrophage populations and their heterogeneity in health and disease.

## Data Availability Statement

The original contributions presented in the study are included in the article/**Supplementary Material**. Further inquiries can be directed to the corresponding authors.

## Ethics Statement

The animal study was reviewed and approved by Sir William Dunn School of Pathology (SWDSOP) ethical review body (AWERB), University of Oxford.

## Author Contributions

AR, DG, and GP designed the research. BD generated the hCD68-CreERT2 line. AR performed experiments with help from GP, AR analyzed the data. All authors contributed to the article and approved the submitted version.

## Funding

DG acknowledges support from the BHF Centre of Regenerative Medicine, Oxford (RM/13/3/30159). This work was funded by British Heart Foundation (BHF) Programme Grant awards to DG and KC (RG/15/10/31485 and RG/17/10/32859) and a BHF Chair Award (CH/16/1/32013) to KC. DG, KC. and G.P are members of the MeRIAD consortium which is supported by a Novo Nordisk Foundation (NNF) grant (NNF15CC0018346) to the University of Oxford, University of Copenhagen (UCPH), and Karolinska Institute. AR is the recipient of a BHF graduate studentship FS/17/68/33478.

## Conflict of Interest

The authors declare that the research was conducted in the absence of any commercial or financial relationships that could be construed as a potential conflict of interest.

## Publisher’s Note

All claims expressed in this article are solely those of the authors and do not necessarily represent those of their affiliated organizations, or those of the publisher, the editors and the reviewers. Any product that may be evaluated in this article, or claim that may be made by its manufacturer, is not guaranteed or endorsed by the publisher.

## References

[1] Wynn TA Chawla A Pollard JW. Macrophage Biology in Development, Homeostasis and Disease. Nat (2013) 496:445–55. doi: 10.1038/nature12034 PMC3725458 23619691

[2] Mosser DM Hamidzadeh K Goncalves R. Macrophages and the Maintenance of Homeostasis. Cell Mol Immunol (2020) 18:579–87. doi: 10.1038/s41423-020-00541-3 PMC7491045 32934339

[3] Wynn TA Vannella KM. Macrophages in Tissue Repair, Regeneration, and Fibrosis. Immunity (2016) 44:450–62. doi: 10.1016/j.immuni.2016.02.015 PMC4794754 26982353

[4] Wentworth JM Naselli G Brown WA Doyle L Phipson B Smyth GK. Pro-Inflammatory CD11c+CD206+ Adipose Tissue Macrophages Are Associated With Insulin Resistance in Human Obesity. Diabetes (2010) 59:1648–56. doi: 10.2337/db09-0287 PMC2889764 20357360

[5] Nathan C Ding A. Nonresolving Inflammation. Cell (2010) 140:871–82. doi: 10.1016/j.cell.2010.02.029 20303877

[6] Barrett TJ. Macrophages in Atherosclerosis Regression. Arterioscler Thromb Vasc Biol (2020) 40:20–33. doi: 10.1161/ATVBAHA.119.312802 31722535 PMC6946104

[7] Hettinger J Richards DM Hansson J Barra MM Joschko A-C Krijgsveld J. Origin of Monocytes and Macrophages in a Committed Progenitor. Nat Immunol (2013) 14:821–30. doi: 10.1038/ni.2638 23812096

[8] Yona S Kim K-W Wolf Y Mildner A Varol D Breker M. Fate Mapping Reveals Origins and Dynamics of Monocytes and Tissue Macrophages Under Homeostasis. Immunity (2013) 38:79–91. doi: 10.1016/j.immuni.2012.12.001 23273845 PMC3908543

[9] Gosselin D Link VM Romanoski CE Fonseca GJ Eichenfield DZ Spann NJ. Environment Drives Selection and Function of Enhancers Controlling Tissue-Specific Macrophage Identities. Cell (2014) 159:1327–40. doi: 10.1016/j.cell.2014.11.023 PMC4364385 25480297

[10] Summers KM Bush SJ Hume DA. Network Analysis of Transcriptomic Diversity Amongst Resident Tissue Macrophages and Dendritic Cells in the Mouse Mononuclear Phagocyte System. PloS Biol (2020) 18:e3000859. doi: 10.1371/JOURNAL.PBIO.3000859 33031383 PMC7575120

[11] Hildreth AD Ma F Wong YY Sun R Pellegrini M O’Sullivan TE. Single-Cell Sequencing of Human White Adipose Tissue Identifies New Cell States in Health and Obesity. Nat Immunol (2021) 22:639–53. doi: 10.1038/s41590-021-00922-4 PMC8102391 33907320

[12] Evren E Ringqvist E Tripathi KP Sleiers N Rives IC Alisjahbana A. Distinct Developmental Pathways From Blood Monocytes Generate Human Lung Macrophage Diversity. Immunity (2021) 54:259–275.e7. doi: 10.1016/j.immuni.2020.12.003 33382972

[13] McCubbrey AL Barthel L Mould KJ Mohning MP Redente EF Janssen WJ. Selective and Inducible Targeting of CD11b+ Mononuclear Phagocytes in the Murine Lung With Hcd68-rtTA Transgenic Systems. Am J Physiol Lung Cell Mol Physiol (2016) 311:L87–L100. doi: 10.1152/ajplung.00141.2016 27190063 PMC4967193

[14] Iwasaki H Akashi K. Myeloid Lineage Commitment From the Hematopoietic Stem Cell. Immunity (2007) 26:726–40. doi: 10.1016/j.immuni.2007.06.004 17582345

[15] Fogg DK Sibon C Miled C Jung S Aucouturier P Littman DR. A Clonogenic Bone Marrow Progenitor Specific for Macrophages and Dendritic Cells. Science (2006) 311:83–7. doi: 10.1126/science.1117729 16322423

[16] Gibbings SL Thomas SM Atif SM McCubbrey AL Desch AN Danhorn T. Three Unique Interstitial Macrophages in the Murine Lung at Steady State. Am J Respir Cell Mol Biol (2017) 57:66–76. doi: 10.1165/rcmb.2016-0361OC 28257233 PMC5516280

[17] Nahrendorf M Swirski FK. Abandoning M1/M2 for a Network Model of Macrophage Function. Circ Res (2016) 119:414–7. doi: 10.1161/CIRCRESAHA.116.309194 PMC4965179 27458196

[18] Xue J Schmidt SV Sander J Draffehn A Krebs W Quester I. Transcriptome-Based Network Analysis Reveals a Spectrum Model of Human Macrophage Activation. Immunity (2014) 40:274–88. doi: 10.1016/j.immuni.2014.01.006 PMC3991396 24530056

[19] Leid J Carrelha J Boukarabila H Epelman S Jacobsen SEW Lavine KJ. Primitive Embryonic Macrophages Are Required for Coronary Development and Maturation. Circ Res (2016) 118:1498–511. doi: 10.1161/CIRCRESAHA.115.308270 PMC5567774 27009605

[20] Cahill TJ Sun X Ravaud C Villa Del Campo C Klaourakis K Lupu I-E. Tissue-Resident Macrophages Regulate Lymphatic Vessel Growth and Patterning in the Developing Heart. Development (2021) 148(3): dev194563. doi: 10.1242/dev.194563 33462113 PMC7875498

[21] Hulsmans M Clauss S Xiao L Aguirre AD King KR Hanley A. Macrophages Facilitate Electrical Conduction in the Heart. Cell (2017) 169:510–522.e20. doi: 10.1016/j.cell.2017.03.050 28431249 PMC5474950

[22] Wong NR Mohan J Kopecky BJ Guo S Du L Leid J. Resident Cardiac Macrophages Mediate Adaptive Myocardial Remodeling. Immunity (2021) 54:2072–2088.e7. doi: 10.1016/j.immuni.2021.07.003 34320366 PMC8446343

[23] Chazaud B. Inflammation and Skeletal Muscle Regeneration: Leave It to the Macrophages! Trends Immunol (2020) 41:481–92. doi: 10.1016/j.it.2020.04.006 32362490

[24] Wang Z Koenig AL Lavine KJ Apte RS. Macrophage Plasticity and Function in the Eye and Heart. Trends Immunol (2019) 40:825–41. doi: 10.1016/j.it.2019.07.002 PMC6719788 31422901

[25] Bou Ghosn EE Cassado AA Govoni GR Fukuhara T Yang Y Monack DM. Two Physically, Functionally, and Developmentally Distinct Peritoneal Macrophage Subsets. Proc Natl Acad Sci USA (2010) 107:2568–73. doi: 10.1073/PNAS.0915000107/SUPPL_FILE/PNAS.200915000SI.PDF PMC2823920 20133793

[26] Holness CL Da Silva RP Fawcett J Gordon S Simmons DL. Macrosialin, a Mouse Macrophage-Restricted Glycoprotein, Is a Member of the Lamp/Lgp Family. J Biol Chem (1993) 268:9661–6. doi: 10.1016/S0021-9258(18)98400-0 8486654

[27] Greaves DR Quinn CM Seldin MF Gordon S. Functional Comparison of the Murine Macrosialin and Human CD68 Promoters in Macrophage and Nonmacrophage Cell Lines. Genomics (1998) 54:165–8. doi: 10.1006/geno.1998.5546 9806844

[28] Gough PJ Gordon S Greaves DR. The Use of Human CD68 Transcriptional Regulatory Sequences to Direct High-Level Expression of Class A Scavenger Receptor in Macrophages *In Vitro* and *In Vivo* . Immunology (2001) 103:351–61. doi: 10.1046/j.1365-2567.2001.01256.x PMC1783239 11454064

[29] Iqbal AJ McNeill E Kapellos TS Regan-Komito D Norman S Burd S. Human CD68 Promoter GFP Transgenic Mice Allow Analysis of Monocyte to Macrophage Differentiation *In Vivo* . Blood (2014) 124:e33–44. doi: 10.1182/blood-2014-04-568691 PMC4192756 25030063

[30] McNeill E Iqbal AJ Jones D Patel J Coutinho P Taylor L. Tracking Monocyte Recruitment and Macrophage Accumulation in Atherosclerotic Plaque Progression Using a Novel Hcd68gfp/ApoE-/- Reporter Mouse-Brief Report. Arterioscler Thromb Vasc Biol (2017) 37:258–63. doi: 10.1161/ATVBAHA.116.308367 PMC5274540 27908893

[31] Park I Goddard ME Cole JE Zanin N Lyytikäinen L-P Lehtimäki T. C-Type Lectin Receptor CLEC4A2 Promotes Tissue Adaptation of Macrophages and Protects Against Atherosclerosis. Nat Commun (2022) 13:215. doi: 10.1038/s41467-021-27862-9 35017526 PMC8752790

[32] Lang R Rutschman RL Greaves DR Murray PJ. Autocrine Deactivation of Macrophages in Transgenic Mice Constitutively Overexpressing IL-10 Under Control of the Human CD68 Promoter. J Immunol (2002) 168:3402–11. doi: 10.4049/JIMMUNOL.168.7.3402 11907098

[33] Metzger D Clifford J Chiba H Chambon P. Conditional Site-Specific Recombination in Mammalian Cells Using a Ligand-Dependent Chimeric Cre Recombinase. Proc Natl Acad Sci USA (1995) 92:6991–5. doi: 10.1073/PNAS.92.15.6991 PMC41457 7624356

[34] Blériot C Ginhoux F. Understanding the Heterogeneity of Resident Liver Macrophages. Front Immunol (2019) 10:2694. doi: 10.3389/fimmu.2019.02694 31803196 PMC6877662

[35] Lu H Howatt DA Balakrishnan A Graham MJ Mullick AE Daugherty A. Hypercholesterolemia Induced by a PCSK9 Gain-Of-Function Mutation Augments Angiotensin II–Induced Abdominal Aortic Aneurysms in C57BL/6 Mice—Brief Report. Arterioscler Thromb Vasc Biol (2016) 36:1753–7. doi: 10.1161/ATVBAHA.116.307613 PMC5001883 27470509

[36] Gordon S Taylor PR. Monocyte and Macrophage Heterogeneity. Nat Rev Immunol (2005) 5:953–64. doi: 10.1038/nri1733 16322748

[37] Guilliams M Mildner A Yona S. Developmental and Functional Heterogeneity of Monocytes. Immunity (2018) 49:595–613. doi: 10.1016/J.IMMUNI.2018.10.005 30332628

[38] Chistiakov DA Killingsworth MC Myasoedova VA Orekhov AN Bobryshev YV. CD68/macrosialin: Not Just a Histochemical Marker. Lab Investig (2016) 97:4–13. doi: 10.1038/labinvest.2016.116 27869795

[39] Amanzada A Ahmed Malik I Blaschke M Khan S Rahman H Ramadori G. Identification of CD68+ Neutrophil Granulocytes in *In Vitro* Model of Acute Inflammation and Inflammatory Bowel Disease. Int J Clin Exp Pathol (2013) 6:561. doi: 10.1055/s-0033-1352657 23573303 PMC3606846

[40] Pillai MM Hayes B Torok-Storb B. Inducible Transgenes Under the Control of the Hcd68 Promoter Identifies Mouse Macrophages With a Distribution That Differs From the F4/80 and CSF-1R Expressing Populations. Exp Hematol (2009) 37:1387. doi: 10.1016/J.EXPHEM.2009.09.003 19772887 PMC2925267

[41] Grabert K Sehgal A Irvine KM Wollscheid-Lengeling E Ozdemir DD Stables J. A Transgenic Line That Reports CSF1R Protein Expression Provides a Definitive Marker for the Mouse Mononuclear Phagocyte System. J Immunol (2020) 205:3154–66. doi: 10.4049/jimmunol.2000835 33139489

[42] Jahn HM Kasakow CV Helfer A Michely J Verkhratsky A Maurer HH. Refined Protocols of Tamoxifen Injection for Inducible DNA Recombination in Mouse Astroglia. Sci Rep (2018) 8:1–11. doi: 10.1038/s41598-018-24085-9 29651133 PMC5897555

[43] Hashimoto D Chow A Noizat C Teo P Beasley MB Leboeuf M. Tissue-Resident Macrophages Self-Maintain Locally Throughout Adult Life With Minimal Contribution From Circulating Monocytes. Immunity (2013) 38:792–804. doi: 10.1016/J.IMMUNI.2013.04.004 23601688 PMC3853406

[44] Kline DL Cliffton EE. LifesPan of Lezlcocytes in Man. J App Physiol (1952) 5(2):79–84. doi: 10.1152/jappl.1952.5.2.79 12990547

[45] Clausen BE Burkhardt C Reith W Renkawitz R Förster I. Conditional Gene Targeting in Macrophages and Granulocytes Using LysMcre Mice. Transgenic Res (1999) 8:265–77. doi: 10.1023/A:1008942828960 10621974

[46] Deng L Zhou JF Sellers RS Li JF Nguyen AV Wang Y. A Novel Mouse Model of Inflammatory Bowel Disease Links Mammalian Target of Rapamycin-Dependent Hyperproliferation of Colonic Epithelium to Inflammation-Associated Tumorigenesis. Am J Pathol (2010) 176:952–67. doi: 10.2353/AJPATH.2010.090622 PMC2808099 20042677

[47] Abram CL Roberge GL Hu Y Lowell CA. Comparative Analysis of the Efficiency and Specificity of Myeloid-Cre Deleting Strains Using ROSA-EYFP Reporter Mice. J Immunol Methods (2014) 408:89–100. doi: 10.1016/j.jim.2014.05.009 24857755 PMC4105345

[48] Ferron M Vacher J. Targeted Expression of Cre Recombinase in Macrophages and Osteoclasts in Transgenic Mice. genesis (2005) 41:138–45. doi: 10.1002/GENE.20108 15754380

[49] Schaller E Macfarlane AJ Rupec RA Gordon S McKnight AJ Pfeffer K. Inactivation of the F4/80 Glycoprotein in the Mouse Germ Line. Mol Cell Biol (2002) 22:8035–43. doi: 10.1128/MCB.22.22.8035-8043.2002 PMC134735 12391169

