# Supplementary material for: A Human CD68 Promoter-Driven Inducible Cre-Recombinase Mouse Line Allows Specific Targeting of Tissue Resident Macrophages
Source: Front Immunol. 2022 Jul 6;13:918636. doi: 10.3389/fimmu.2022.918636 (PMC9298978; doi:10.3389/fimmu.2022.918636)
Supplement: Supplementary file 1 [file DataSheet_1.pdf]

Supplementary Material

1 Supplementary Figures and Tables

1.1 Supplementary Figures

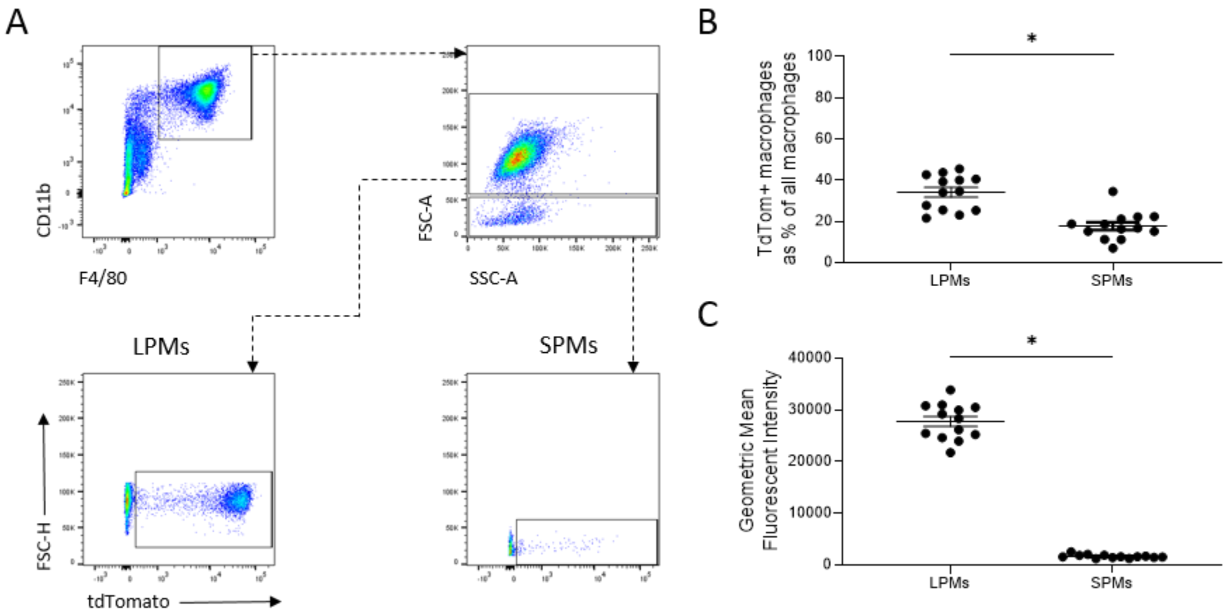

Supplementary Figure 1: tdTomato expression varies between large and small peritoneal macrophages. A) Representative dot plots of the gating strategy for large peritoneal macrophages (LPMs) and small peritoneal macrophages (SPMs). **B)** Frequency of tdTomato-expressing large and small peritoneal macrophages as a proportion of all macrophages of the given subtype with corresponding mean fluorescent intensity in **(C)**.

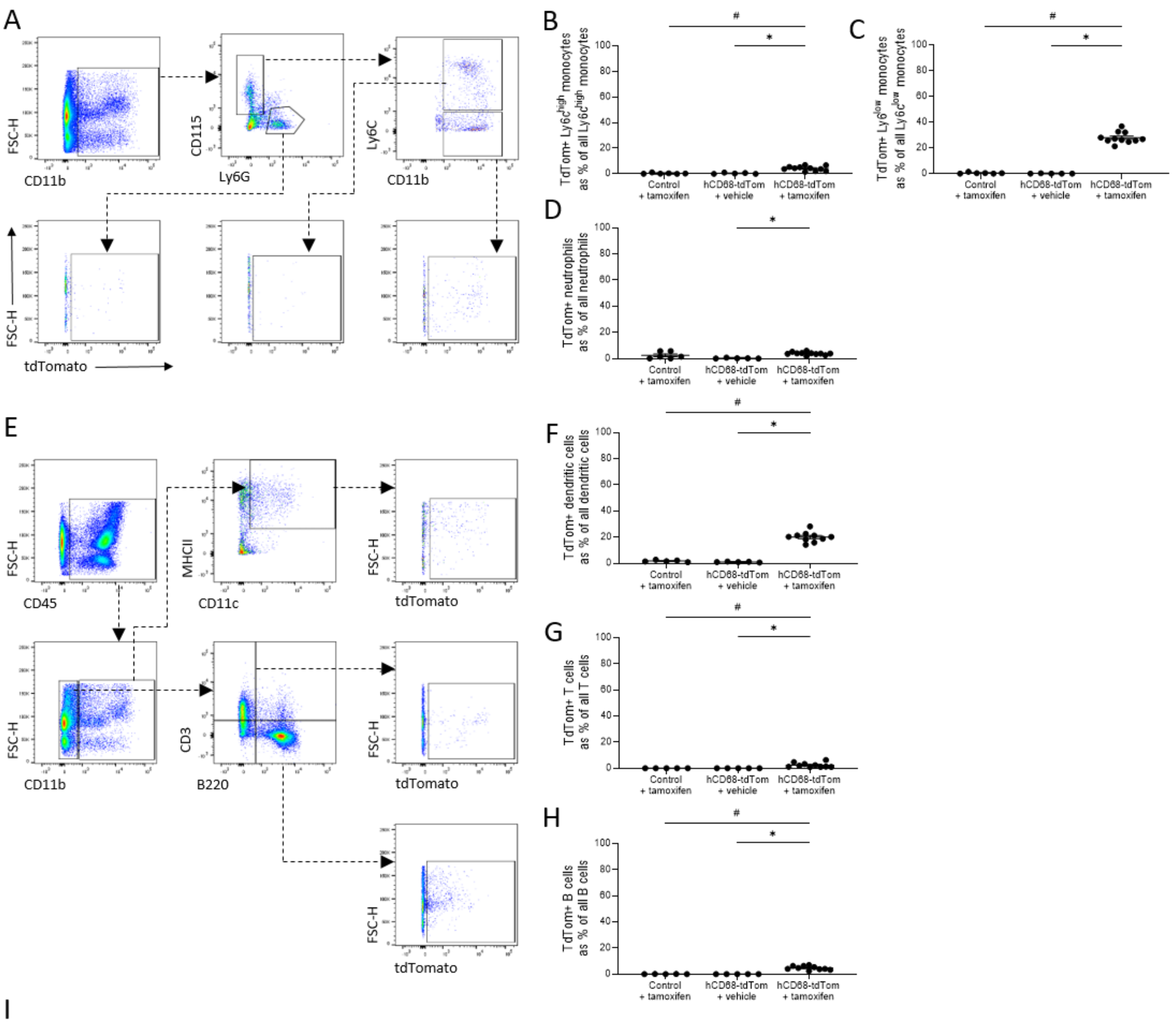

I

Population	Control + tamoxifen	hCD68-tdTom + vehicle	hCD68-tdTom + tamoxifen
Ly6C^{high} monocytes	0.39 ± 0.17	0.39 ± 0.20	4.25 ± 0.54*#
Ly6C^{low} monocytes	0.45 ± 0.22	0.07 ± 0.07	27.91 ± 1.30*#
Neutrophils	2.42 ± 1.06	0.22 ± 0.13	3.86 ± 0.34*
Dendritic cells	1.78 ± 0.33	1.03 ± 0.13	20.08 ± 1.21*#
T cells	0.03 ± 0.005	0.03 ± 0.003	2.40 ± 0.58*#
B cells	0.07 ± 0.02	0.03 ± 0.003	4.72 ± 0.47*#

Supplementary Figure 2: TdTomato expression in spleen non-macrophage populations. A) Representative dot plots of the gating strategy for splenic neutrophils (CD11b$^+$, CD115$^-$, Ly6G$^+$), Ly6C^{high} monocytes (CD11b$^+$, CD115$^+$, Ly6G$^-$, Ly6C$^+$) and Ly6C^{low} monocytes (CD11b$^+$, CD115$^+$, Ly6G$^-$, Ly6C$^-$). **B)** Frequency of tdTomato-expressing Ly6C^{high} monocytes as a proportion of all Ly6C^{high} monocytes. **C)** Frequency of tdTomato-expressing Ly6C^{low} monocytes as a proportion of all Ly6C^{low} monocytes. **D)** Frequency of tdTomato-expressing neutrophils as a proportion of all

neutrophils. **E)** Representative dot plots of the gating strategy for splenic dendritic cells ($CD45^+$, $CD11b^+$, $MHCII^+$, $CD11c^+$), T cells ($CD45^+$, $CD11b^-$, $CD3^+$, $B220^-$) and B cells ($CD45^+$, $CD11b^-$, $CD3^-$, $B220^+$). **F)** Frequency of tdTomato-expressing dendritic cells as a proportion of all dendritic cells. **G)** Frequency of tdTomato-expressing T cells as a proportion of all T cells. **H)** Frequency of tdTomato-expressing B cells as a proportion of all B cells. **I)** Summary table of tdTomato expression in all splenic non-macrophage populations represented as mean ± SEM; one-way ANOVA (# = $p<0.05$ for 'Control + tamoxifen' vs 'hCD68-tdTom + tamoxifen'; *= $p<0.05$ for 'hCD68-tdTom + vehicle' vs 'hCD86-tdTom + tamoxifen').

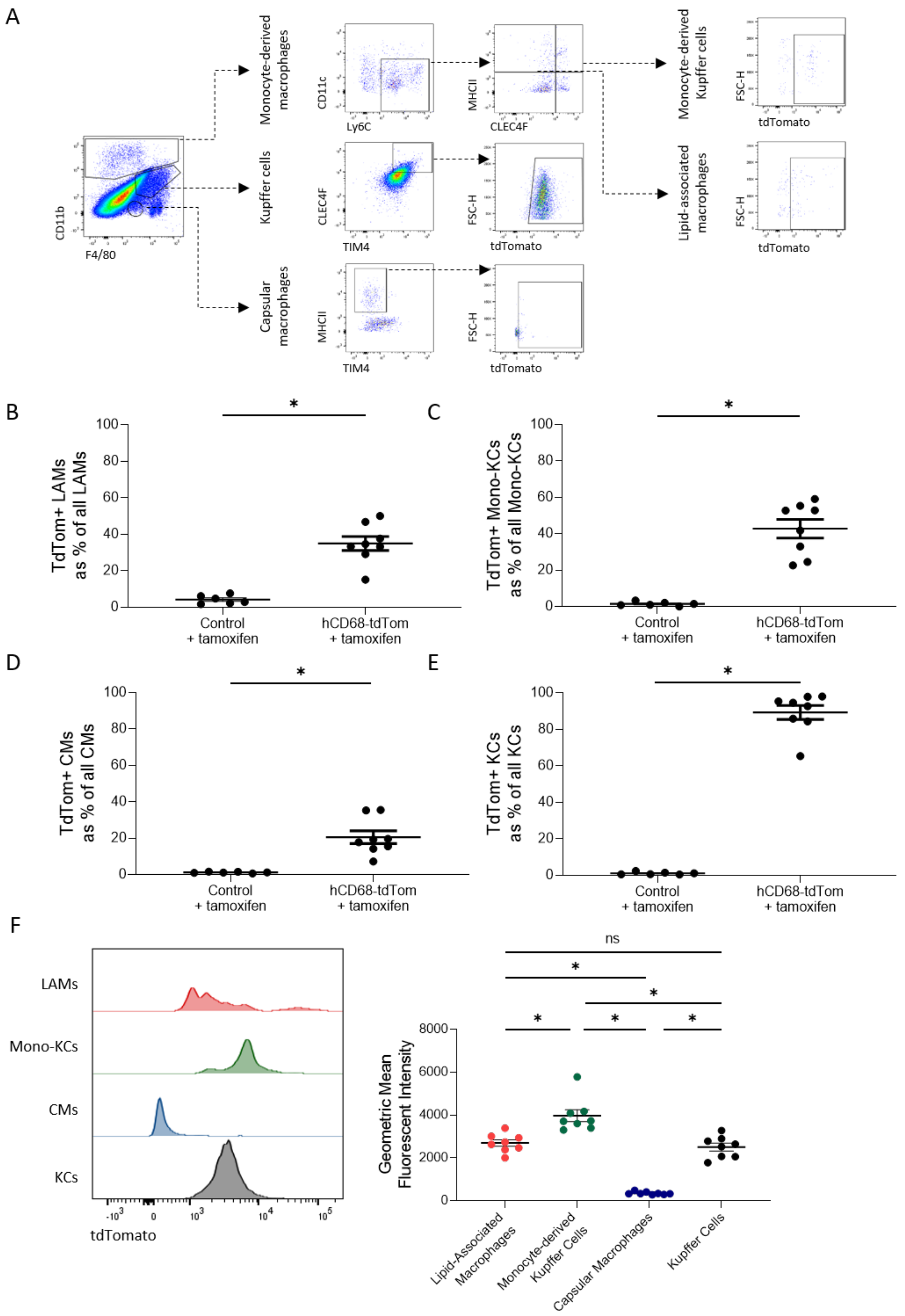
A
Monocyte-derived macrophages
Kupffer cells
Capsular macrophages
CD11b
F4/80
CD11c
Ly6C
MHCII
CLEC4F
CLEC4F
TIM4
MHCII
TIM4
FSC-H
tdTomato
Monocyte-derived Kupffer cells
Lipid-associated macrophages
B
TdTom+ LAMs as % of all LAMs
C
TdTom+ Mono-KCs as % of all Mono-KCs
D
TdTom+ CMs as % of all CMs
E
TdTom+ KCs as % of all KCs
Control + tamoxifen
hCD68-tdTom + tamoxifen
*
F
LAMs
Mono-KCs
CMs
KCs
tdTomato
Geometric Mean Fluorescent Intensity
ns
Lipid-Associated Macrophages
Monocyte-derived Kupffer Cells
Capsular Macrophages
Kupffer Cells

Supplementary Figure 3: hCD68 promoter drives the expression of TdTomato in different macrophage sub-populations in the liver to a varying degree. A) Representative dot plots of the gating strategy for liver monocyte-derived macrophages ($CD11b^{high}$, $F4/80^{low}$, $CD11c^{-}$, $Ly6C^{+}$) sub-divided into monocyte-derived Kupffer cells (Mono-KCs, $MHCII^{+}$, $CLEC4F^{high}$) and lipid-associated macrophages (LAMs, $MHCII^{+}$, $CLEC4F^{low}$), Kupffer cells (KCs, $CD11b^{int}$, $F4/80^{high}$, $CLEC4F^{+}$, $TIM4^{+}$) and capsular macrophages (CMs, $CD11b^{-}$, $F4/80^{+}$, $MHCII^{+}$, $TIM4^{-}$). **B)** Frequency of tdTomato-expressing lipid-associated macrophages as a proportion of all lipid-associated macrophages. **C)** Frequency of tdTomato-expressing monocyte-derived Kupffer cells as a proportion of all monocyte-derived Kupffer cells. **D)** Frequency of tdTomato-expressing capsular macrophages as a proportion of all capsular macrophages. **E)** Frequency of tdTomato-expressing Kupffer cells as a proportion of all Kupffer cells. **F)** Histogram of the mean fluorescent intensity of liver macrophage sub populations with corresponding values graphed and colour-coded on the right; lipid-associated macrophages are represented by red histogram, monocyte-derived Kupffer cells as green, capsular macrophages as blue and Kupffer cells as black, one-way ANOVA or student t-test as appropriate, (* = $p<0.05$).

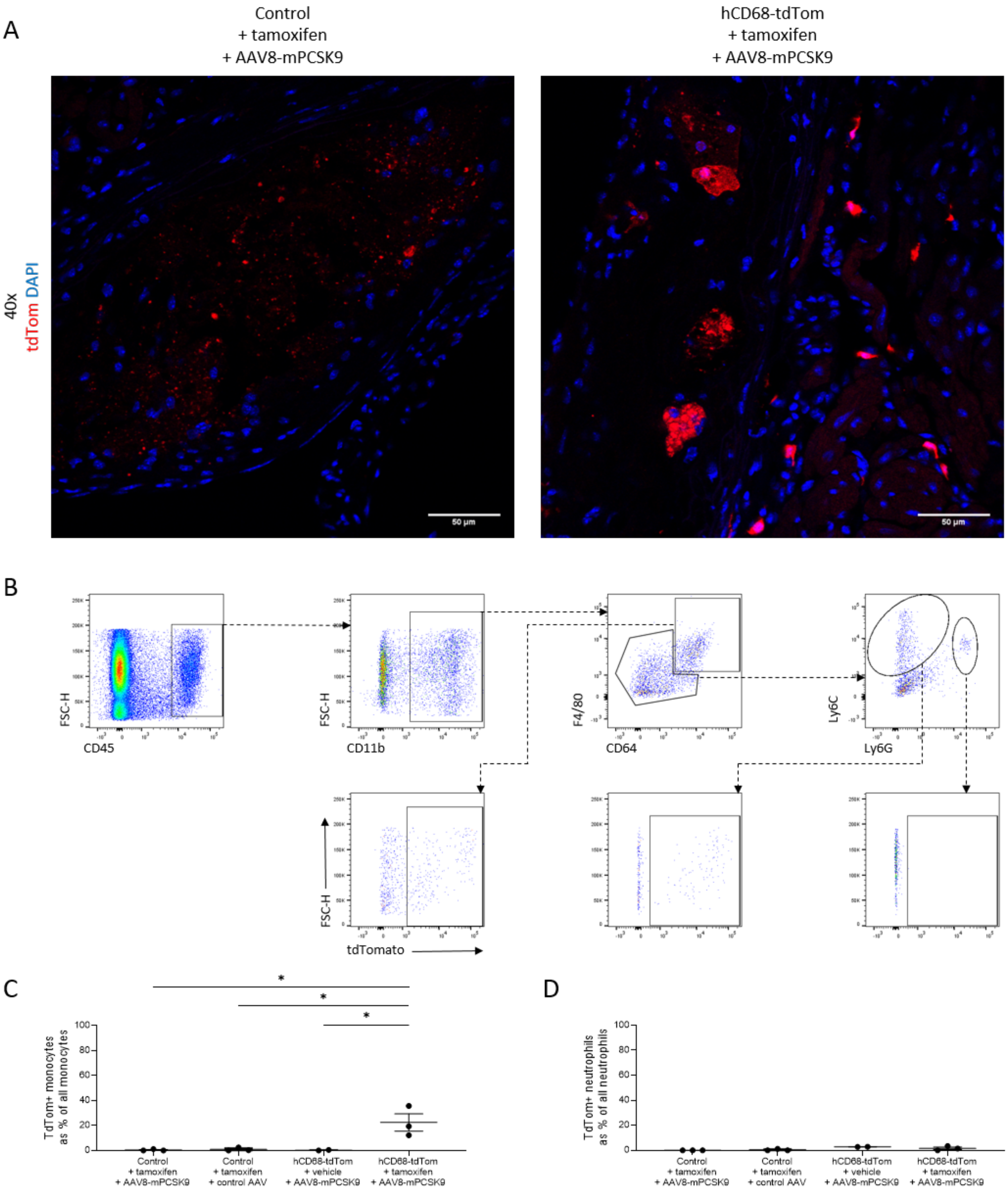
A
Control
+ tamoxifen
+ AAV8-mPCSK9
hCD68-tdTom
+ tamoxifen
+ AAV8-mPCSK9
40x tdTom DAPI
50 µm
50 µm
B
FSC-H
CD45
FSC-H
CD11b
F4/80
CD64
Ly6C
Ly6G
FSC-H
tdTomato
C
TdTom+ monocytes as % of all monocytes
Control + tamoxifen + AAV8-mPCSK9
Control + tamoxifen + control AAV
hCD68-tdTom + vehicle + AAV8-mPCSK9
hCD68-tdTom + tamoxifen + AAV8-mPCSK9
D
TdTom+ neutrophils as % of all neutrophils
Control + tamoxifen + AAV8-mPCSK9
Control + tamoxifen + control AAV
hCD68-tdTom + vehicle + AAV8-mPCSK9
hCD68-tdTom + tamoxifen + AAV8-mPCSK9

Supplementary Figure 4: hCD68 promoter driven activation of TdTom expression in non-macrophage myeloid cells in the aorta. A) Representative immunofluorescence images of endogenous tdTomato expression and auto-fluorescent lipid droplets in the atherosclerotic plaques at 40x magnification with DAPI in blue and tdTomato in red; scale bar = 50 µm. **B)** Representative dot plots of the gating strategy for aortic macrophages ($CD45^+$, $CD11b^+$, $F4/80^+$, $CD64^+$), monocytes ($CD45^+$, $CD11b^+$, $F4/80^-$, $CD64^-$, $Ly6C^+$, $Ly6G^-$) and neutrophils ($CD45^+$, $CD11b^+$, $F4/80^-$, $CD64^-$, $Ly6C^+$, $Ly6G^+$). **C)** Frequency of tdTomato-expressing aortic monocytes as a proportion of all aortic monocytes. **D)** Frequency of tdTomato-expressing aortic neutrophils as a proportion of all aortic neutrophils; one-way ANOVA (* = $p<0.05$).

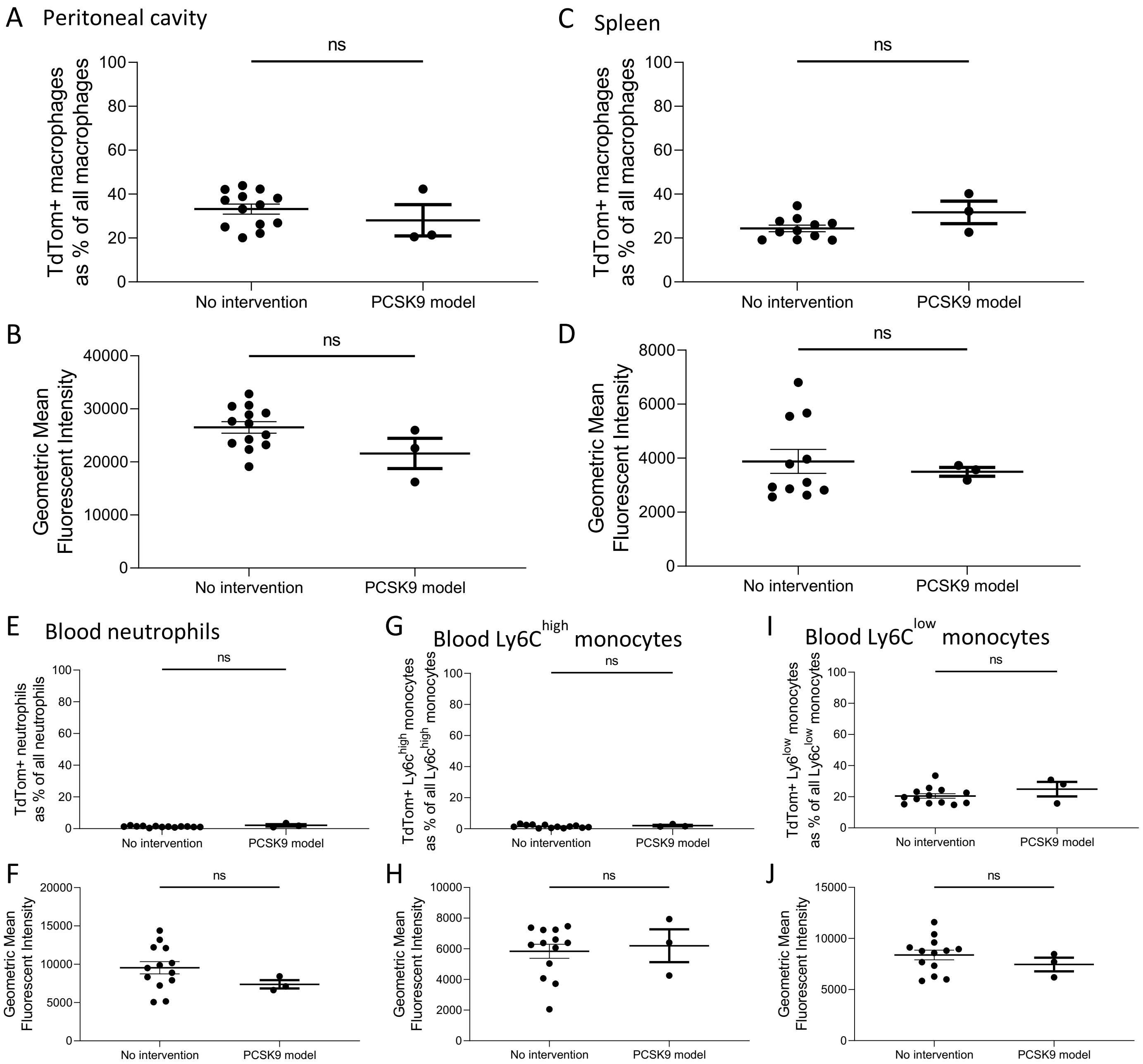

Supplementary Figure 5: Inducible PCSK9 model of atherosclerosis does not change the efficiency of Cre recombinase activation in hCD68-tdTom mice. hCD68-tdTom mice were orally gavaged with tamoxifen for 5 days and tissues collected after 5 days induction period either without any pathological interventions (‘No intervention’ group) or after injection with PCSK9 viral vector and 16 weeks of high fat diet (‘PCSK9 model’ group). Frequency of tdTomato-expressing macrophages as a proportion of all macrophages in (A) peritoneal cavity and (C) spleen and with respective mean fluorescence intensity values of tdTomato expression in the TdTom-expressing macrophage population in (B) and (D). Frequency of tdTomato-expressing blood (E) neutrophils, (G) Ly6C^{high} monocytes and (I) Ly6C^{low} monocytes as a proportion of all blood leukocytes of the given type for ‘no intervention’ and ‘PCSK9 model’ with respective mean fluorescence intensity values of tdTomato expression in the TdTom-expressing cells of the given type in (F), (H) and (J); student t-test.

1.2 Supplementary Tables

Supplementary Table 1: tdTomato expression in spleen non-macrophage populations. Percentage of tdTomato-expressing leukocyte populations in the blood represented as mean ± SEM; one-way ANOVA (* = p<0.05 for hCD68-tdTom+vehicle+PCSK9 vs. hCD68-tdTom+tamoxifen+PCSK9).

Population	Control +tamoxifen +control AAV	Control +tamoxifen +PCSK9	hCD68-tdTom +vehicle +PCSK9	hCD68-tdTom +tamoxifen +PCSK9
$Ly6C^{high}$ monocytes	0.05 ± 0.05	0.09 ± 0.04	0.17 ± 0.07	2.10 ± 0.51
$Ly6C^{low}$ monocytes	0.04 ± 0.03	0.00	0.00	25.00 ± 4.70
Neutrophils	0.08 ± 0.04	0.148 ± 0.06	0.08 ± 0.01	2.10 ± 0.73
Dendritic cells	0.61 ± 0.61	0.10 ± 0.09	0.00	5.4 ± 1.3
$CD4^+$ T cells	0.86 ± 0.31	1.9 ± 0.30	1.1 ± 0.16	3.9 ± 0.59
$CD8^+$ T cells	0.63 ± 0.37	0.33 ± 0.07	0.24 ± 0.04	1.5 ± 0.49
B cells	0.19 ± 0.02	0.14 ± 0.05	0.20 ± 0.08	0.55 ± 0.17

Supplementary Table 2: tdTomato expression in spleen non-macrophage populations. Percentage of tdTomato-expressing leukocyte populations in the spleen represented as mean ± SEM; one-way ANOVA (* = p<0.05 for hCD68-tdTom+vehicle+PCSK9 vs. hCD68-tdTom+tamoxifen+PCSK9).

Population	Control +tamoxifen +control AAV	Control +tamoxifen +PCSK9	hCD68-tdTom +vehicle +PCSK9	hCD68-tdTom +tamoxifen +PCSK9
Ly6C^{high} monocytes	0.93 ± 0.19	0.59 ± 0.12	1.31 ± 0.39	13.50 ± 2.69
Ly6C^{low} monocytes	0.82 ± 0.38	1.20 ± 0.26	1.87 ± 0.18	35.10 ± 8.98
Neutrophils	0.37 ± 0.13	0.32 ± 0.09	0.25 ± 0.11	3.17 ± 0.98
Dendritic cells	3.210 ± 0.07	2.920 ± 0.38	2.635 ± 0.02	27.47 ± 5.57
T cells	0.08 ± 0.01	0.12 ± 0.02	0.10 ± 0.03	2.98 ± 1.22
B cells	0.10 ± 0.03	0.1083 ± 0.01	0.1350 ± 0.01	3.877 ± 0.52

Supplementary Table 3: tdTomato expression in spleen non-macrophage populations. Percentage of tdTomato-expressing splenic immune non-macrophage populations represented as mean ± SEM; student t-test (* = p<0.05 for 'No intervention' vs 'PCSK9 model' groups as defined in Supplementary Figure 4).

Population	No intervention	PCSK9 model
Ly6C^{high} monocytes	4.25 ± 0.54	13.50 ± 2.69*
Ly6C^{low} monocytes	27.91 ± 1.30	35.10 ± 8.98
Neutrophils	3.86 ± 0.34	3.18 ± 0.98
Dendritic cells	20.08 ± 1.21	27.47 ± 5.57
T cells	2.18 ± 0.45	2.98 ± 1.22
B cells	4.72 ± 0.47	3.88 ± 0.52
